# Immunogenic Consensus Sequence T helper Epitopes for a Pan-*Burkholderia* Biodefense Vaccine

**DOI:** 10.4172/1745-7580.1000043

**Published:** 2011-05

**Authors:** Anne S. De Groot, Matthew Ardito, Leonard Moise, Eric A. Gustafson, Denice Spero, Gloria Tejada, William Martin

**Affiliations:** 1 EpiVax 146 Clifford St, Providence, RI 02903, USA; 2 Institute for Immunology and Informatics, University of Rhode Island, 80 Washington St., Providence, RI 02903, USA

## Abstract

**Background:**

Biodefense vaccines against Category B bioterror agents *Burkholderia pseudomallei* (BPM) and *Burkholderia mallei* (BM) are needed, as they are both easily accessible to terrorists and have strong weaponization potential. *Burkholderia cepaciae* (BC), a related pathogen, causes chronic lung infections in cystic fibrosis patients. Since BPM, BM and BC are all intracellular bacteria, they are excellent targets for T cell-based vaccines. However, the sheer volume of available genomic data requires the aid of immunoinformatics for vaccine design. Using EpiMatrix, ClustiMer and EpiAssembler, a set of immunoinformatic vaccine design tools, we screened the 31 available *Burkholderia* genomes and performed initial tests of our selections that are candidates for an epitope-based multi-pathogen vaccine against *Burkholderia* species.

**Results:**

Immunoinformatics analysis of 31 *Burkholderia* genomes yielded 350,004 9-mer candidate vaccine peptides of which 133,469 had perfect conservation across the 10 BM genomes, 175,722 had perfect conservation across the 11 BPM genomes and 40,813 had perfect conservation across the 10 BC genomes. Further screening with EpiMatrix yielded 54,010 high-scoring Class II epitopes; these were assembled into 2,880 longer highly conserved ‘immunogenic consensus sequence’ T helper epitopes. 100% of the peptides bound to at least one HLA class II allele *in vitro*, 92.7% bound to at least two alleles, 82.9% to three, and 75.6% of the binding results were consistent with the immunoinformatics analysis.

**Conclusions:**

Our results show it is possible to rapidly identify promiscuous T helper epitopes conserved across multiple *Burkholderia* species and test their binding to HLA ligands *in vitro*. The next step in our process will be to test the epitopes *ex vivo* using peripheral leukocytes from BC, BPM infected humans and for immunogenicity in human HLA transgenic mice. We expect that this approach will lead to development of a licensable, pan-*Burkholderia* biodefense vaccine.

## Background

Due to their exceptionally high virulence in animals and humans, and their potential for weaponization as aerosols, BP and BPM are both classified as category B bio-threat agents. In addition to use as a countermeasure, *Burkholderia* vaccines would also contribute to improving human health in certain patient populations (such as immunocompromised patients) and sectors of the globe (such as Thailand) most affected by exposure to these pathogens.

Attempts to develop both whole-cell killed and live attenuated vaccines against *Burkholderia* species, have failed to result in a complete protective immune response in mice. Ulrich et al. developed two differently attenuated strains of *B. mallei* (a capsule-negative mutant and a branched-chain amino acid auxotroph) to protect against aerosolized *B. mallei* challenge. No protection was observed to the capsule-negative mutant, but the auxotroph conferred a slight protective advantage although the mice did not clear the infection [1]. Other vaccine targets include the capsular polysaccharides and LPS, as there is significant genetic and structural conservation between the capsular polysaccharides of these species [2]. Recently, subunit vaccines against BC have shown promise. Mice nasally immunized with *Burkholderia multivorans* outer membrane proteins rapidly resolved pulmonary infections following *B. multivorans* challenge and also elicited cross-protection against *B. cenocepacia* [3, 4]. Although *B. cepaciae* proteins that appear to be protective have been identified, no vaccine against BC currently exists [5]. To date, no vaccine for any pathogenic *Burkholderia* species is approved for human use.

Although antibodies can protect against severe infection by BM, passive prophylaxis has not been shown to confer sterilizing protective immunity. This is likely due to *Burkholderia’s* capability of latent long-term intracellular infections. Cell-mediated immune response, in conjunction with a humoral response, may be required to successfully protect against infection with *Burkholderia* species, and to clear intracellular infections. In general, it is believed that robust cell-mediated immune responses to *Burkholderia* will be required for an effective protective or therapeutic vaccine [6].

Evidence for protective cellular immune response to BPM infection comes from several live attenuated vaccine studies in mice and suggests that cell-mediated immunity is critical. Immunization of C57BL/6 mice with a mutant of BPM (*aroC*) deficient in aromatic amino acid synthesis resulted in sterile immunity [7]. BALB/c mice inoculated with a BPM transposable 2D2 insertion mutant (*ilvl*) auxotrophic for branched chain amino acids induced a protective response and 85% survived a lethal wild type BPM challenge. However, BPM persisted in spleen, liver, kidney and lung tissues up to 30 days post challenge [8]. Splenic BPM-specific T cells, detected in immunized mice, proliferated and produced interferon-gamma *in vitro* in response to dead bacteria. Assessment of T cell antigen specificity indicated that subpopulations of BPM-specific T cells were responsive to secreted proteins. Adoptive immunization of severe combined immunodeficiency mice with T cells from 2D2 live-attenuated BPM mutant-immunized mice resulted in increased survival compared to naïve T cell recipients. This suggests that 2D2 immunization can generate T cell-mediated immunity [9]. CD4+ and CD8+ cell depletion studies argue that CD4+ cells, but not CD8+ cells, mediated this protection *in vivo*.

Cell mediated immune response to antigens produced by live organisms are important to protection from *Burkholderia*. In a separate study, immune responses and resistance following subcutaneous immunization with live BPM were compared with exposure to heat-killed culture filtrate and sonicated BPM antigens. Compared to heat-killed BPM, significant protection was generated in BALB/c mice following exposure to live bacteria. Thus, CD4+ T cells can mediate vaccine-induced immunity to experimental melioidosis [9]. These studies suggest CD4+ T cell recognition of processed and secreted proteins from live bacteria are crucial for disease protection. These results also suggest that the type of immune response generated *in vivo* is influenced by the nature of the BPM antigens, and that immune responses to those proteins that are actively secreted may be required to stimulate a protective immune response [10].

T cell epitopes are critical mediators of cellular immunity and are derived from a pathogen‘s proteins via two pathways. In one, a protein derived from an intracellular pathogen is processed and its constituent peptides bind to major histocompatability complex (MHC) Class I molecules. Alternatively, proteins derived from pathogens external to the antigen presenting cells (APCs) are processed in the proteolytic compartment; these constituent peptides bind to MHC Class II molecules. After processing and binding, MHC Class I and Class II peptide complexes are then transported to the surface of an APC, where they are exposed to interrogation by passing T cells (CD8+ and CD4+ T cells, respectively). From these different antigen processing and presentation pathways, two different T cell responses are generated: a CD4+ T helper immune response and a CD8+ cytotoxic lymphocyte immune response. After initial exposure to pathogen (or vaccine), memory T cells are established that respond more rapidly and efficiently upon subsequent exposure.

We have previously used this genome-derived epitope-based vaccine design approach to develop a prototype *Francisella tularensis* Type A (subsp. *tularensis*) vaccine that confers 60% protection against heterologous lethal respiratory challenge with the live vaccine strain (LVS), an attenuated subsp. *holarctica* derivative [11, 12]. To our knowledge no subunit vaccine for tularemia has achieved a comparable level of protection in this well-developed lethal respiratory challenge model in HLA transgenic mice. In parallel studies, we developed an epitope-based vaccine composed of T cell epitopes derived from sequences conserved between vaccinia and variola. This vaccine was 100% protective against intra-nasal small pox challenge in HLA transgenic mice and occurred in the absence of detectable antibody response [13]. Seven poxvirus genomes were previously the maximum number submitted for analysis by our vaccine design tools. Here we employed the same approach to a much larger set of genomic sequences, with the goal of selecting the optimal sequences for a vaccine that could protect against multiple *Burkholderia* species.

## Methods

We utilized bioinformatics and immunological tools to identify candidate proteins from 31 *Burkholderia* genomes for inclusion in a multipathogen-specific prophylactic vaccine as previously published [11, 13, 14]. Details on the approach used for the multipath vaccine are provided below. We then used T cell epitope mapping tools (Conservatrix, EpiMatrix) to identify 9-mer amino acid sequences that were both highly conserved in *Burkholderia* genomes and potentially immunogenic. These putative epitopes were then assembled into immunogenic consensus sequence clusters (using EpiAssembler) and their *in vitro* binding properties tested against 5 human class II HLA alleles. We then hand-selected the best 70 clusters, of which 41 were synthesized for further testing in soluble HLA binding assays as previously described [11, 13].

### Genome Collection

Genomes from 31 strains of *Burkholderia* were obtained from Pathema, a proprietary genome database from the J. Craig Venter Institute (http://pathema.jcvi.org/cgi-bin/Burkholderia). These included protein-coding genomes from 10 *B. mallei* strains (FMH: 5600 ORFS; NCTC10229: 5519 ORFS; 2002721280: 5519 ORFS; ATCC10399: 5746 ORFS; ATCC23344: 5229 ORFS; GB8: 5936 ORFS; JHU: 5559 ORFS; NCTC10247: 5869 ORFS; PRL-20: 5469 ORFS; and SAVP1: 5200 ORFS), 11 *B. pseudomallei* strains (1106a: 7180 ORFS; 1106b: 7223 ORFS; 1655: 6908 ORFS; 1710a: 7540 ORFS; 406e: 6866 ORFS; 576: 7400 ORFS; 668: 7135 ORFS; K96243: 6304 ORFS; MSHR346: 7588 ORFS; PAS-TEUR52237: 7140 ORFS; S13: 7253 ORFS), 2 *B. ambifaria* strains (AMMD: 6976 ORFS; MC40-6: 7163 ORFS), 2 *B. cenocepacia* strains (AU1054: 7109 ORFS; HI2424: 7227 ORFS), 5 *B. multivorans* strains (ATCC17616-JGI: 6779 ORFS; ATCC17616-Tohoku: 6699 ORFS; CGD1: 6572 ORFS; CGD2: 6653 ORFS; CGD2M: 6646 ORFS) and 1 *B. vietnamiensis* strain. *B. ambifaria*, B*. cenocepacia*, *B. multivorans* and *B. vietnamiensis* (G4: 8423 ORFS) comprise the *Burkholderia cepaciae* complex group [15].

### Genome Alignment and Cross-walk

In order to identify proteins conserved within various *Burkholderia* species, the *B. mallei*, *B. pseudomallei* and *B. cepaciae* strains were aligned using GB8, MSHR346 and G4 as reference genomes, respectively. These intra species conserved proteins were then analyzed for inter-species conservation using a comparative genomics tool from Pathema (http://pathways.jcvi.org/comp-genomics). This identified proteins in each of the three reference genomes that have hits (defined as any two proteins with a sequence identity greater than or equal to 80%) among the selected comparison genomes.

### Secretion Analysis and Conservatrix

**Figure 1:**
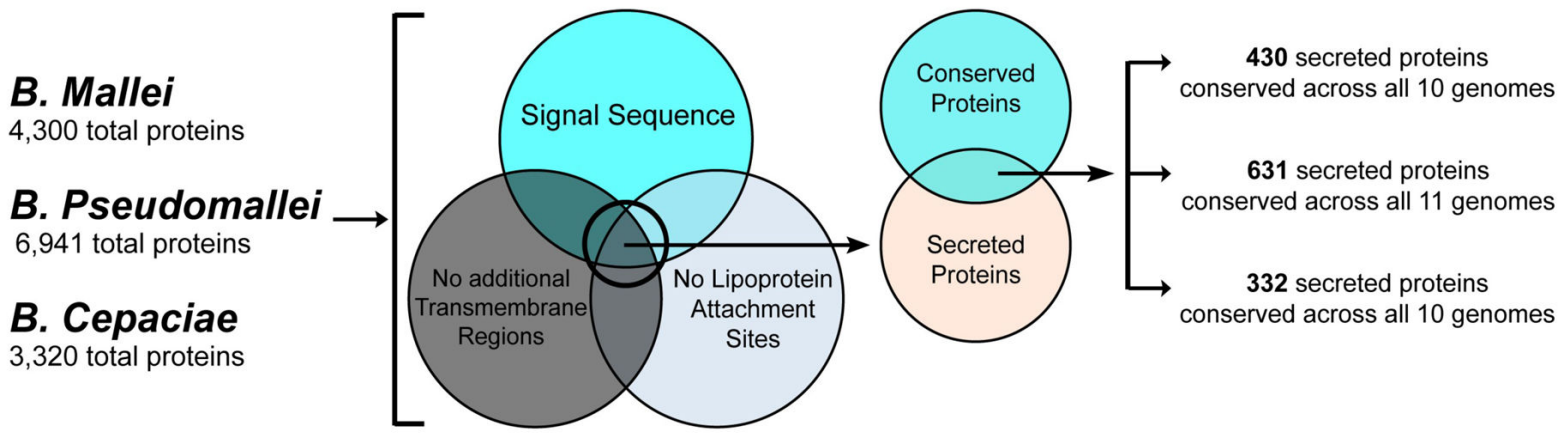
Selection of conserved and secreted *Burkholderia* proteins Proteins containing a signal sequence, lacking lipoprotein attachment sites and lacking predicted transmembrane domains were analyzed for conservation across 3 *Burkholderia* reference genomes (*B. mallei* GB8, *B. pseudomallei* MSHR346 and *B. cepaciae* G4).

The Phobius program was used to identify single peptides and transmembrane segments and the LipoP program was used to identify lipoprotein attachment sites in proteins from each of the 31 *Burkholderia* genomes (http://phobius.sbc.su.se/; http://www.cbs.dtu.dk/services/LipoP/) [16, 17]. Proteins with a signal sequence, no lipoprotein attachment sites and no more than 1 transmembrane segment were selected for further analysis (Figure 1). In order to target functionally or structurally important epitopes that are conserved between *Burkholderia* species, the Conservatrix algorithm parsed input sequences into component strings, typically comprised of overlapping 9-mer segments, then searched the input database for matching segments found in at least two of the three *Burkholderia* species and ultimately produced a sequence conservation frequency table for each 9-mer.

### EpiMatrix Analysis

**Figure 2:**
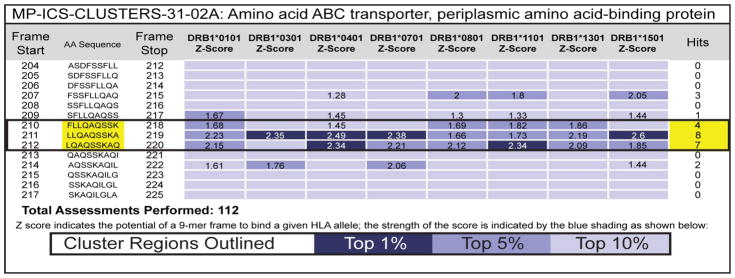
EpiBar located on peptide MP-ICS-CLUSTERS-31-02A EpiMatrix analysis of the BPM amino ABC transporter, periplasmic amino acid-binding protein (GenBank ID# 237814370) identified residues 210–220 within the MP-ICS-CLUSTERS-31-02A peptide as an immunogenic EpiBar. High Z-scores (above 1.64) across 4 or more human class II MHC alleles are considered hits and constitute an EpiBar.

EpiMatrix, a matrix-based epitope-mapping algorithm, was used to identify Class II HLA epitopes from the conserved 9-mer peptides identified. Potential binding of the 9-mer sequences was scored for 8 common HLA alleles that cover >90% of the human population (DRB1*0101, DRB1*0301, DRB1*0401, DRB1*0701, DRB1*0801, DRB1*1101, DRB1*1301 and DRB1*1501) [18, 19]. While assessment scores (Z-scores) range from approximately −3 to +3, Z-scores equal to or greater than 1.64 are generally comprise the top 5% of any given peptide set, are defined as “Hits” and considered potentially immunogenic. Z-scores above 2.32 are in the top 1% and are extremely likely to bind MHC molecule. A 9-mer frame predicted to react to at least 4 different HLA alleles is considered an EpiBar. EpiBars may be the signature feature of highly immunogenic, promiscuous class II epitopes (Figure 2); in previously published studies we have observed that these epitopes tend to be more immunogenic than epitopes that do not contain EpiBars [11–13].

### EpiAssembler and Blastimer

**Figure 3:**
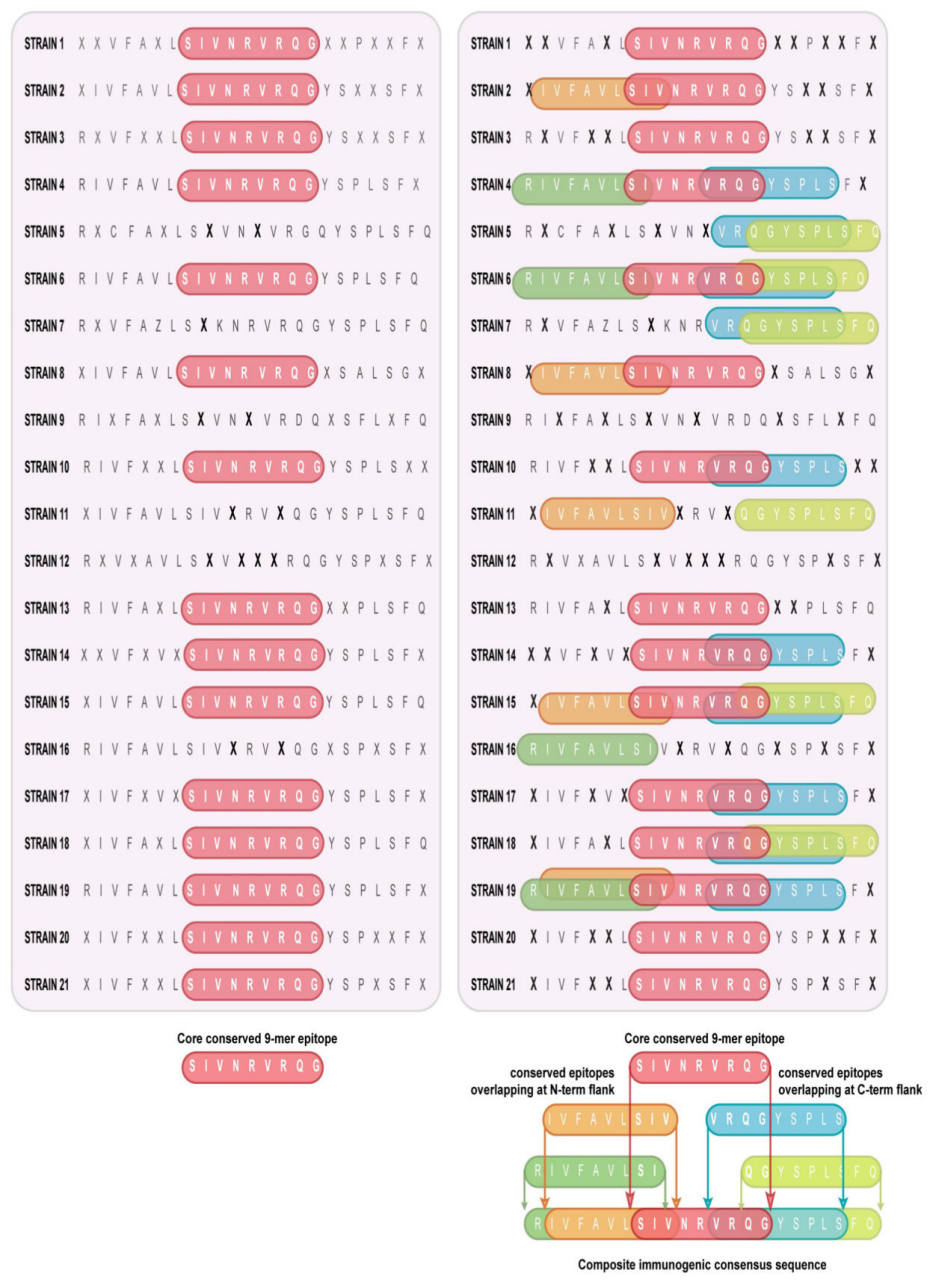
Constructing an Immunogenic Consensus Sequence (A) EpiAssembler identified a core conserved 9-mer epitope (red) and identified naturally overlapping N- and C-terminal flanking regions from other 9-mer epitopes (orange, green and blue) in a serial fashion to generate a composite immunogenic consensus sequence.

EpiAssembler was then used to identify sets of overlapping and conserved epitopes from selected 9-mer peptides, as well as assemble them into extended immunogenic consensus sequences (ICS) [20]. This algorithm iteratively identifies core highly conserved sequences that contain multiple putative T cell epitopes (clusters) and extends the core sequence right and left culling from a database of similarly highly conserved, putatively epitope rich sequences (Figure 3). The EpiMatrix scores within these ICS clusters are then aggregated to create an EpiMatrix Cluster Immunogenicity Score. As cross-reactivity with self may lead to deleterious immune responses, we evaluated the ICS clusters for homology to the human genome by BLAST analysis [21]. Peptides sharing greater than 70% identity with sequences in the human genome were eliminated from consideration. None of the 2,880 *Burkholderia* ICS clusters were found to have significant homology (>90%) to the human genome.

### ICS Selection and Peptide Synthesis

In order to minimize technical difficulties with peptide synthesis and low water solubility stemming from hydrophobic peptides, the amino acid hydropathy score was assessed for each ICS cluster by GRAVY [22]. Each ICS sequence was constructed to contain a minimal set of T cell epitopes, as well as cover a maximum number of observed *Burkholderia* strains. This was accomplished by comparing the remaining ICS clusters for cross-species conservation. Selected epitopes were synthesized, purified by HPLC and verified by mass spectrometry (21^st^ Century Biochemicals, Marlboro, MA).

### Class II HLA binding assay

Purified, soluble HLA Class II DR competition binding assays were performed as previously described [23]. Briefly, non-biotinylated ICS peptides over a wide range of concentrations competed with biotinylated influenza hemagglutinin 306–318 standard peptide (0.1 M) for binding to purified DRB1*0101, DRB1*0301, DRB1*0401, DRB1*0701, and DRB1*1501 (50 nM) in 96-well plates for 24 hours at 37°C. ELISA plates coated with pan anti-Class II antibodies (L243, anti-HLA-DR; BioXCell, West Lebanon, NH) were blocked with 5% FBS in PBS-0.05% Tween-20 and then bound to the DR/ peptide complexes for 1 hour at 37°C. Following extensive washing in PBS-0.05% Tween-20, the ELISA plates were developed by addition of streptavidin-europium and analyzed on a Victor^3^V Microtiter Plate Reader. Percent inhibition and IC_50_ values of the biotinylated peptide binding were calculated using SigmaPlot 11.1 software.

## Results

### *In silico* epitope mapping

#### Conservatrix, EpiMatrix

Comparative genomic alignment analysis of the *B. mallei* GB8, *B. pseudomallei* MSHR346 and *B. cepaciae* G4 genomes as references yielded a total of 3,288 proteins conserved across all 10 genomes of *B. mallei*, 4,682 proteins conserved across all 11 genomes of *B. pseudomallei* and 2,823 proteins conserved across all 10 genomes of *B. cepaciae*. LipoP and Phobius analyses identified 10,793 secreted core ORFs. Conservatrix analysis of these ORFs yielded 350,004 9-mer peptides; of which 133,469 had perfect conservation across the 10 BM genomes, 175,722 had perfect conservation across the 11 BPM genomes and 40,813 had perfect conservation across the 10 BC genomes. EpiMatrix analyses of these conserved *Burkholderia* 9-mer peptides yielded 54,010 putative Class II HLA epitopes.

#### EpiAssembler

Using the 54,010 unique 9-mer peptides as a starting point, EpiAssembler produced 2,880 candidate ICS clusters. Figure 3 shows a conceptual example of ICS assembly from conserved and overlapping HLA peptide epitopes using EpiAssembler.

#### Blastimer

Cross-conservation with the human genome may lead to deleterious anti-self immune responses to vaccines. Therefore, we used BLAST analysis to confirm that none of these ICS clusters had any significant homology to the human genome. GRAVY analysis removed 19 ICS clusters with extremely hydrophobic properties. Cross-species conservation analysis yielded 90 ICS epitopes >70% conserved between *B. mallei* and B*. pseudomallei*, 42 ICS clusters >70% conserved between *B. mallei* and *B. cepaciae*, 32 ICS clusters >70% conserved between *B. pseudomallei* and *B. cepaciae* and 20 ICS clusters >70% conserved among all 3 *Burkholderia* species.

#### Protein ontology

**Figure 4:**
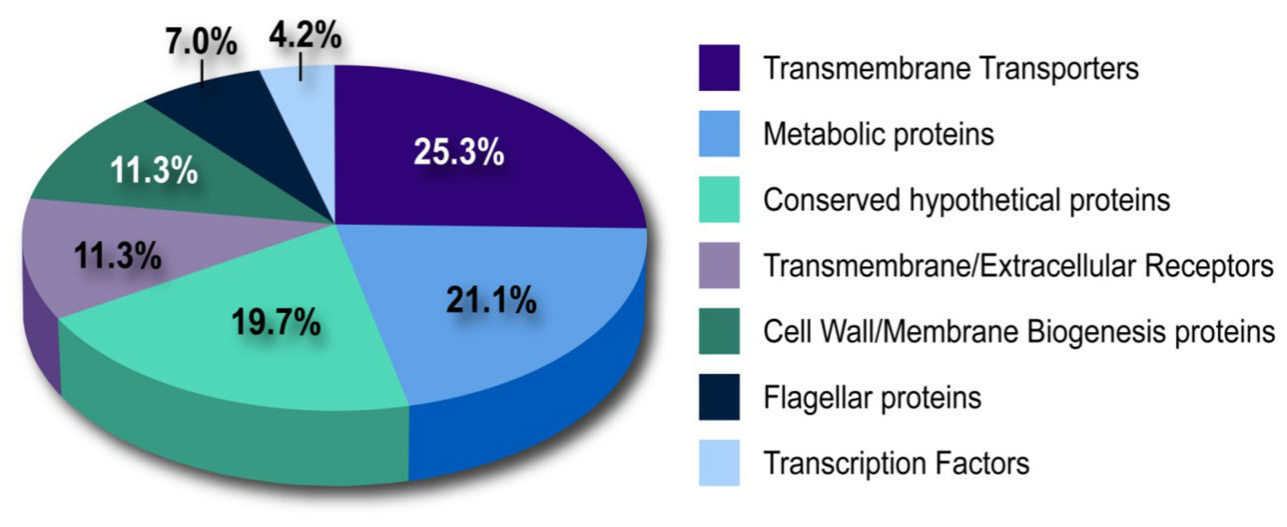
Functional classification of identified immunogenic consensus sequence cluster source proteins Functional categories are based on cellular biological processes ascertained from manual gene ontology analysis using the UniProt protein database.

The top-scoring 70 Class II ICS clusters were selected for further analysis (Table 1). These ICS cluster peptides indeed correspond to *Burkholderia* proteins predicted to have a variety of cellular functions (Figure 4). Many of these proteins, such as transmembrane transporters, transmembrane and extracellular receptors, cell wall and membrane biogenesis proteins and flagellar proteins, are predicted to function at the bacterial cell surface, even though they passed the initial screen for putative secreted proteins. In at least four cases, transcription factors have been identified as secreted proteins [24–28], thus while rare, this is not an unprecedented observation. Based on the previous examples, secretion of these proteins may be indicative of a highly immunogenic protein, therefore we have elected to retain these epitopes in our vaccine development program.

#### BLAST against other bacterial proteins

A BLAST search was performed for the epitopes identified in this manner against non-human, bactetrial protein. No hits were identified that had greater than 70% conservation (six out of nine amino acid residues conserved). Therefore, these epitopes are relatively unique and unlikely to be cross reactive with other commensals and other pathogens. Vaccination with these epitopes would be expected to drive a pan-*Burkholderia* immune response; that will be the focus of the next stage of our gene-to-vaccine program.

### Class II HLA-binding analyses

**Figure 5:**
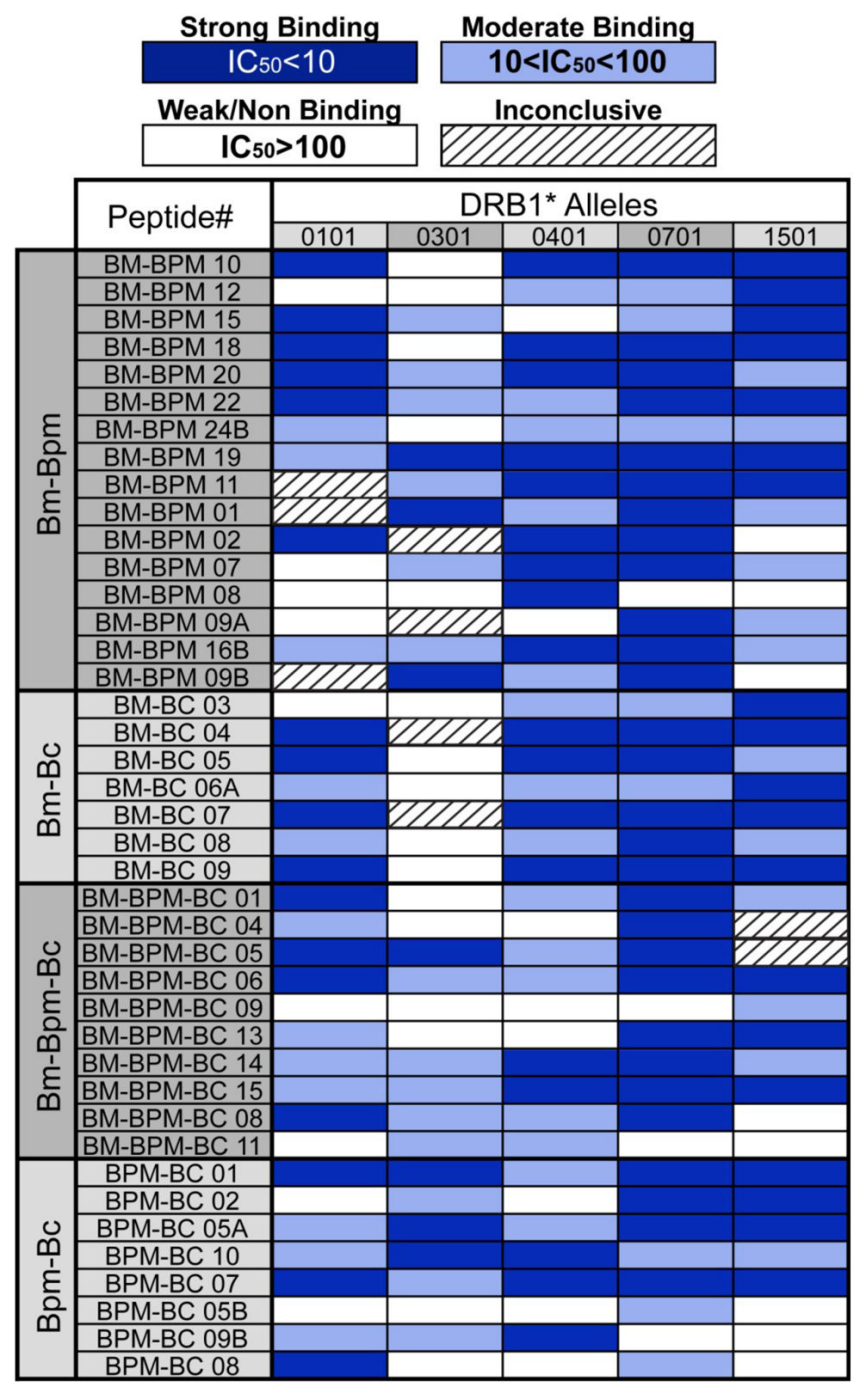
MHC Class II HLA-binding analysis for promiscuity Column 1: Inter-*Burkholderia* species conservation of immunogenic consensus sequence (ICS) peptides (*B. mallei* = BM, *B. pseudomallei* = BPM, *B. cepaciae* = BC); column 2: ICS peptide ID; columns 3–7: ICS peptide binding affinities to the human HLA class II alleles DRB1*0101, DRB1*0301 DRB1*0401 DRB1*0701 DRB1*1501. Weak or no affinity (IC_50_>100 M = white); moderate affinity (100 M >IC_50_>10 M = light blue); strong affinity (IC_50_<10 M = dark blue); inconclusive binding (hash).

Peptide binding affinities for HLA DRB*0101, DRB1*0301, DRB1*0401, DRB1*0701, and DRB1*1501 were determined in competitive binding assays. Of the 205 ICS peptide-HLA binding interactions assayed, 44% displayed strong binding (IC_50_<10), 30% showed moderate binding (10<IC_50_<100) and 22% displayed weak or non-binding (IC_50_>100). In only 9 cases, the HLA binding results were inconclusive (Figure 5).

All peptides bound to at least one of the HLA alleles for which they were predicted, 92.7% bound to two alleles for which they were predicted, 82.9% bound to three alleles for which they were predicted. These data support the use of this approach for the high-volume genomic screening for vaccine candidates. Therefore, we proceeded to the next step in our development process with this highly conserved, highly promiscuous candidate epitope cohort.

**Figure 6:**
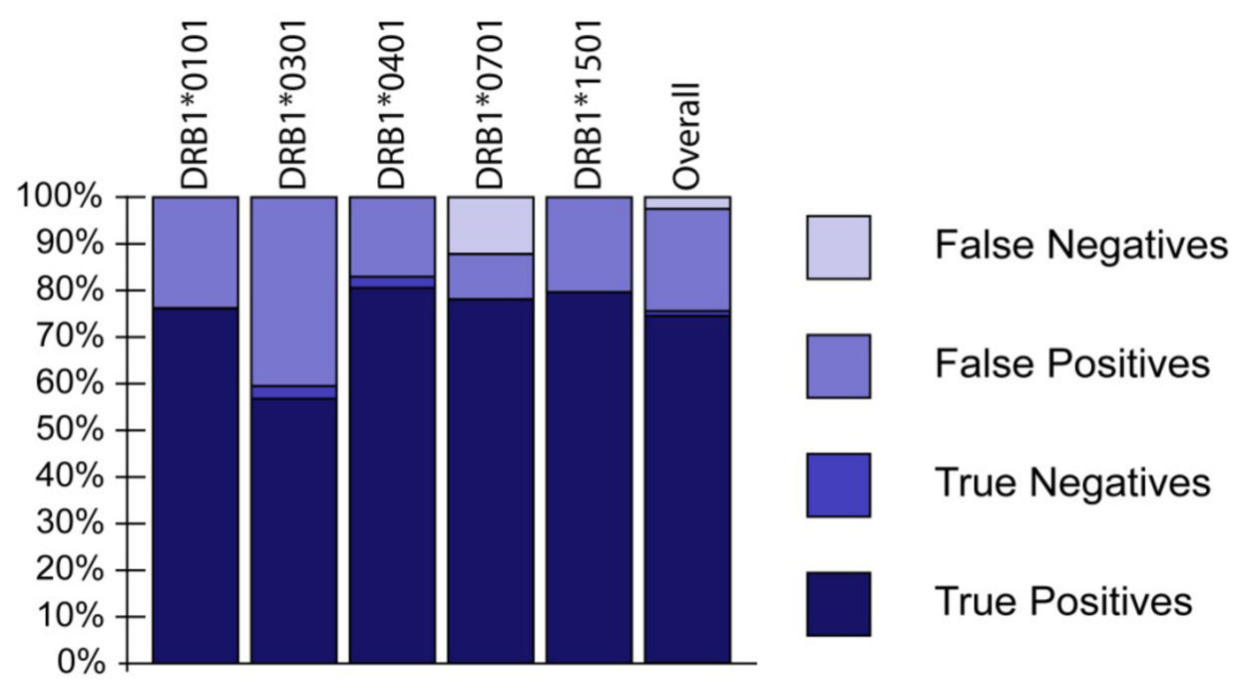
EpiMatrix binding prediction success The HLA class II binding result for each ICS peptide was compared to its EpiMatrix predictive binding scores for each human HLA class II allele. True positives (dark blue) reflect correctly predicted HLA-binding peptide results. False positives (medium blue) reflect incorrectly predicted HLA-binding peptide results. True negatives (light blue) reflect correctly predicted non-HLA-binding peptide results. False negatives (grey) indicate incorrectly predicted non-HLA-binding peptide results.

Comparison between computational predictions and actual *in vitro* HLA binding results show 75.6% overall predictive success rate when excluding inconclusive results (Figure 6). Epitope prediction success was also compared for each class II MHC allele. Successful binding prediction was 76.3% for DRB1*0101, 59.5% for DRB1*0301, 82.9% for DRB1*0401, 78.6% for DRB1*0701 and 79.5% for DRB1*1501. A lack of accord between positive binding predictions and actual binding data was observed at 23.7% for DRB1*0101, 40.5% for DRB1*0301, 17.1% for DRB1*0401, 9.5% for DRB1*0701 and 20.5% for DRB1*1501 (Figure 6). This could be due to the affinity of the competitor peptide (bound too tightly to compete off), peptide synthesis, problems with peptide aggregation in the *in vitro* assay, or lack of predictive accuracy by the EpiMatrix tool. In a large, retrospective comparison of the EpiMatrix with other online tools, EpiMatrix was as accurate or more accurate than other available epitope prediction tools [29]. Therefore, it is likely that much of the discrepancy between predictions and HLA binding is due to physical interference in the *in vitro* assay.

## Discussion

Using publically accessible bioinformatics tools we identified secreted proteins conserved between 31 different *Burkholderia* genomes and used our validated vaccine design toolkit to select highly conserved class II epitope clusters as potential T cell epitopes for a T cell directed vaccine. These peptides were then evaluated for their *in vitro* binding properties to 5 different human class II HLA alleles.

*Burkholderia mallei* (BM) and *Burkholderia pseudomallei* (BPM) are responsible for the severe diseases glanders and melioidosis, respectively. *Burkholderia mallei* is a Gram-negative, non-motile bacillus that requires a mammalian host environment for survival (Whitlock et al., 2007). BM is the etiological agent of glanders in donkeys, mules, horses and occasionally humans. Horses are the predominant natural reservoir for BM and transmission to humans occurs through direct contact with infected animals [30]. While BM is generally confined to animal species, it can cause severe respiratory infection when aerosolized and for that reason is considered, along with BPM, a Category B pathogen by the NIAID Biodefense Research Agenda [31].

*Burkholderia pseudomallei,* the etiological agent of melioidosis, is a Gram-negative, facultatively anaerobic, motile bacillus that is responsible for a broad spectrum of illnesses in both humans and animals. The incidence of disease is particularly high in Southeast Asia. In Thailand, an estimated 20% of community-acquired septicemias and approximately 40% of deaths due to complications associated with bacterial sepsis can be attributed to this organism [32, 33]. Antibiotic therapy is the first line of defense post-exposure but faces significant challenges. Despite extensive antibiotic regimens, recurrence of infection ranges from 13% to 26% and therapy choice is limited by antibiotic resistance [32, 34, 35]. As a result, mortality rates as high as 50% in northeast Thailand and ~20% in Northern Australia have been observed [32, 34, 36]. However, infection with this pathogen in tropical regions of the world may be underreported. Recrudescence may occur: reactivation of latent BPM in Vietnam veterans up to 18 years after their last exposure has been reported [37].

*Burkholderia cepaciae* (BC) is a Gram-negative, non-sporulating motile bacillus found in a variety of both aquatic and terrestrial environments [38]. BC is an opportunistic human pathogen associated with life-threatening pulmonary infections in immunocompromised individuals and individuals with cystic fibrosis [39].

Based on their highly infectious properties in aerosol form and extremely high virulence, BPM and BM are both classified as category B bioterrorist agents. There is currently no vaccine available for any *Burkholderia* species. Due to the potential bioterrorism threat, the development of a safe and effective *Burkholderia* vaccine is a national and worldwide goal. Addition of BC sequences may contribute to the development of a vaccine that could prevent disease in select cystic fibrosis patient populations in the United States.

Conventional vaccines using whole killed, whole protein, or live attenuated have offered success for over a century. However, development of a *Burkholderia* vaccine through this approach has proven elusive. Inactivated whole cell vaccines provided some *Burkholderia* protection in mouse models, but protection in intravenously challenged mice and sterile immunity was unsuccessful [10, 40–42]. Furthermore, killed, whole-cell BM vaccines did not protect the vaccinated mice from a live challenge (>300 50% lethal doses), suggesting that proteins or polysaccharides that are produced by live bacteria are critically important to protection from BPM and BM disease [43]. BPM vaccine studies using live attenuated virus, killed virus and adoptive immunization provide evidence for CD4+ T cell-mediated vaccine-induced immunity to melioidosis [9]. Despite this progress in vaccine development, *Burkholderia’s* propensity for latent infections along with the undefined mechanistic nature behind several attenuated *Burkholderia* strains pose significant challenges towards developing vaccines approved for human use. Contemporary immunome-derived vaccines have a significant advantage over conventional vaccines; the careful selection of the vaccine components through the use of computer-driven analysis should diminish undesired side effects as those observed with whole pathogen and protein subunit vaccines.

This study couples the current boon of genomic resources with our sophisticated bioinformatics and immunoinformatic tools to design candidate peptide epitopes for a multi-species *Burkholderia* vaccine. This approach moves away from whole protein, killed whole cell and attenuated pathogen-based *Burkholderia* vaccines for several reasons. Potentially dangerous cross-reactive or inert space-consuming epitopes present in canonical vaccines are not included in the vaccine. By eliminating superfluous components, epitope-based vaccines maximize their immunogenic payload as well as maximize the protective efficacy to direct a broad based immune response against multiple antigenic proteins associated with the pathogen(s) and also reduce formulation challenges and cost. Safety concerns stemming from the use of intact recombinant proteins that may have undesired biological activities, such as enzymes, immunomodulators, cross-reactivity or toxins, may also be mitigated through targeted epitope approach. These bioinformatics sequence analysis tools, epitope-mapping tools, microarrays and high-throughput immunology assays successfully identified the minimal essential vaccine components for smallpox, tularemia, *Helicobacter pylori* and tuberculosis vaccines [11–13]. As described here, we are also using this approach for the development of a vaccine for biodefense against multiple *Burkholderia* species. The tools enabling these vaccine development successes are described here, and the anticipated clinical development of immunome-derived and epitope-driven vaccines will be the subject of future reports.

Our results show it is possible to identify and *in vitro* validate T cell epitopes that are conserved across multiple *Burkholderia* species. These epitopes will be further tested in human PBMC and transgenic mice. We aim to use these epitopes for inclusion and further testing in a multi-pathogen-specific *Burkholderia* vaccine. We anticipate that a multi-epitope construct could be administered with an anti-LPS vaccine, already in clinical trial [44], resulting in an effective vaccine directed at providing both humoral and cellular immune response. The resulting multi-pathogen *Burkholderia* vaccine will benefit both the developing world and biodefense.

## Figures and Tables

**Table 1:** Class II Epitopes selected for HLA-binding analyses Column 1: Immunogenic consensus sequence peptide cluster ID; column 2: amino acid sequence for each peptide; column 3: protein description for the parent protein from GenBank; columns 4–6: GenBank ID reference numbers for the reference genomes *B. mallei* (BM) GB8, *B. pseudomallei* (BPM) MSHR346 and *B. cepaciae* (BC) G4.

CLUSTER LABEL	SEQUENCE	SOURCE PROTEIN	GenBank ID		
			BM(GB8)	BPM (MSHR346)	BC(G4)
BM-BC-02	GNRALLLLVLAAISLMASRGE	polyhydroxyalkanoate depolymerase	67643796	237811476	134296512
BM-BPM-07	LKSVLKMVKVYLAVKRRLQPGD	DNA-directed RNA polymerase, beta subunit	67640298	237814156	134294435
BM-BPM-BC-03	YVERVNVLKLNLALDAAQAALA	K+-transporting ATPase, C subunit	67639435	237811537	134296470
BM-BPM-01	AFNFVRQFSLNLRADLIGA	oxidoreductase, short-chain dehydrogenase/reductase family protein	67643921	237813600	134294849
BM-BC-03	FAHLKSVLKVLSAFNPSVKGK	BC nitrate/sulfonate/bicarbonate transporter, periplasmic ligand binding protein	67641562	237814431	134294167
BM-BPM-03	KGEWKLYLHSIRRLLRIDRPGF	Beta-N-acetylhexosaminidase, chitobiase	238561344	237810743	134297015
BM-BPM-10	PMGVYSKRVNSLAALRARPF	D-methionine ABC transporter, periplasmic D-methionine-binding protein	67642900	237813281	134295093
BM-BPM-05	SKLIFSVNSVLAYQAAQQ	cystine-binding periplasmic protein FliY, putative amino acid ABC transporter	238563032	237508413	134293667
BM-BPM-06	ALVLNVLNAALANNVTLD	glycosy hydrolase family protein	67639146	237812240	134295413
BM-BPM-BC-05	ALMSILQAMKAAGALRAAAL	flagellar P-ring protein	238562528	237810465	134297194
BM-BC-04	SGIFRGIKRYFSANPPLAKLS	N-acetylmuramoyl-L-alanine amidase AmiC	238561654	237811107	134296748
BM-BPM-22	MRQFQSIVAQLRAKPLHLM	intracellular PHB depolymerase	67640207	237812617	134288123
BM-BPM-12	VIPWQMGIVAKLLHVMNSGGFKMK	oxidoreductase, short chain dehydrogenase/reductase family	67641011	237810610	134297108
BM-BPM-18	LEQWLRGVKANLARKATGG	ferric iron uptake ABC transporter (FeT) family, periplasmic iron-binding protein	67639460	237811665	134292266
BPM-BC-03	RTALQRSRNLVSIRILSLAL	penicillin-binding protein, 1A family	67642044	237812726	134294482
BM-BPM-19	GSMVASFVLMRLSSVTHTT	ricin-type beta-trefoil lectin domain/galactose oxidase domain protein	238562996	237508926	134296706
BM-BC-05	LPMYTGLHGANRLASNSLLEMA	L-aspartate oxidase	238562659	237811178	134296677
BM-BPM-09b	ARGLVYADGILLSNLLGSSYSMT	TonB-dependent receptor	238562879	237813367	134295041
BM-BPM-15	SKASKQALNLLVNAKVPTRRA	Phospholipase D (PLD) family protein	67642027	237812695	134295648
BM-BPM-11	TEPFSAFINVLAHPSAVMI	Glutathione-binding protein GsiB	67642278	237509488	134293406
BM-BPM-BC-01	KYNDRLSLRRAQAVKSYLV	ompA family protein	238561689	237813303	134295071
BM-BPM-02	ANSMNAFRTNRSLSAAHL	hypothetical protein BURPS1655_A2260	67641082	237811791	134295372
BM-BPM-20	LTRYNRSFFYALAVYQLG	lytic murein transglycosylase B	67642905	237813286	134295088
BM-BPM-BC-04	VCDKVLSQAKALLAKAG	conserved hypothetical protein	238563606	237811018	134293794
BPM-BC-05a	QRHLQRMFKVYLNVSPTHYYR	transcriptional regulator, AraC family protein	238563940	237510183	134292187
BPM-BC-01	KIYEMLLAYRIERALTKDQI	penicillin-binding protein, 1A family	238562280	237814104	134294482
BM-BPM-09a	QAGFRFQHVSNAGILLS	lipid A 3-O-deacylase (PagL) superfamily	238561336	237810753	134297010
BM-BC-06a	NEFFVPLNVRVQKARRVGAAL	N-acetylmuramoyl-L-alanine amidase	238561654	237811107	134296748
BM-BC-07	RNMWQSHRIKLIALSAFTLAE	conserved hypothetical protein	67643124	237814304	134294271
BM-BPM-08	LFDVSALLNQMRRNPSAYGLST	GDSL-like lipase/acylhydrolase domain/outer membrane autotransporter barrel domain protein	67642947	237812898	134293351
BPM-BC-08	NPFMMQQALNKLEGKPV	carboxyl-terminal protease	238563023	237810682	134297049
BM-BPM-13	RDQVLIRFANRSCSVLVM	ATP-dependent dead/deah box RNA helicase DbpA	67639524	237510128	134292614
BPM-BC-02	LRTLEELARLVRLSQRHLQRLQ	transcriptional regulator, AraC family	238563940	237510183	134292187
BPM-BC-07	QEQFKLLLIRTYSGALAQ	ABC transporter, periplasmic ligand binding protein, toluene tolerance TtgeD-like protein	67641601	237814073	134294509
BM-BPM-BC-06	AAAIKYLEALRVELRPA	oxidoreductase, short chain dehydrogenase/reductase family	67641011	237810610	134297108
BM-BPM-BC-09	HHRIYYRLHKLAKQWHAQV	Phosphate regulon sensor protein PhoR	67641079	237811788	134295369
BM-BPM-BC-08	VTTYLKAVQAAGSTDSLLV	putative amino acid uptake ABC transporter, periplasmic amino acid-binding protein	238561553	237814370	134294218
BM-BPM-16b	RRRFDVSIALSSLAGGI	conserved hypothetical protein	238561938	237810872	134295646
BPM-BC-10	YGVIKLNWVILEENVLRQR	mannitol ABC transporter, periplasmic mannitol-binding protein	67641206	237811073	134296800
BM-BPM-BC-11	NGNVSSLGNAKSLRGGTL	flagellar basal body P-ring protein (Flgl)	238562528	237810465	134297194
BM-BC-08	HPMLYYRALTQGLNQTLA	tRNA delta(2)-isopentenylpyrophosphate transferase	67642140	237813667	134294835
BM-BPM-BC-13	NKILRAFLKSKGVKDS	putative amino acid ABC transporter, periplasmic amino acid-binding protein	67644003	237813471	134294953
BM-BPM-BC-14	LQNFYVLTRYNRSADVKRFI	lytic murein transglycosylase B	67642905	237813286	134295088
BM-BC-09	DYIYQKLLGVRGVFA	Tol-Pal system beta propeller repeat protein Tol	238561025	237813605	134294844
BM-BPM-24b	ARGFILGLANAGGD	branched-chain amino acid ABC transporter	238561553	237814370	134294218
BM-BPM-BC-15	TLRIRGLHNAANALAAVS	UDP-N-acetylmuramoyl-L-alanyl-D-glutamate synthetase	238561283	237813921	134294645
BM-BPM-04a	NAAFNFNNAIRFTSVFP	outer membrane porin protein	238561395	237509809	134295282
BPM-BC-05b	GTAIYYRLHKLAKQWHAQV	phosphate regulon sensor kinase PhoR	67641079	237811788	134295369
BPM-BC-09b	AEVALQRSLPAPAAH	tetratricopeptide repeat protein	238561327	237810775	134296995
BM-BPM-21	NVCQLRVSRNALASIAVL	putative lipoprotein, parallel beta-helix repeat protein	67642171	237811603	134296638
MP-ICS-CLUSTERS-31-02A	ASDFSSFLLQAQSSKAQILGLA	ABC branched-chain amino acid family transporter	238561553	237814370	134294218
MP-ICS-CLUSTERS-31-03	GEPRVNVLKLNLALDAAQAAH	K+-transporting ATPase, C subunit (kdpC)	67639435	237811537	134296470
MP-ICS-CLUSTERS-31-01	KKNFTAMLRLDHNRALSQLAA	malate dehydrogenase	67642757	237508279	134293103
MP-ICS-CLUSTERS-31-06	TQTLANMLANLGISINNGSANG	flagellar P-ring protein Flgl (flgl)	238562528	237810465	134297194
MP-ICS-CLUSTERS-31-02B	RAAAKVILNARTGSIVMNQ	flagellar P-ring protein (flgl)	238562528	237810465	134297194
MP-ICS-CLUSTERS-31-04B	SNPYKLDVAKAKALLAKAG	ABC transporter, periplasmic substrate-binding protein	238563343	237508894	134293794
MP-ICS-CLUSTERS-31-05	PRSYFRGLNFLLNEQP	tetratricopeptide repeat protein	67642911	237813292	134295082
MP-ICS-CLUSTERS-31-12	LERFGRYHATLSPGLNIV	SPFH domain protein/band 7 family protein	67641689	237812784	134296009
MP-ICS-CLUSTERS-31-11B	ALNDRLMLAYLTKNAQLPLRPG	amino acid ABC transporter, periplasmic amino acid-binding protein	67640271	237812103	134295624
MP-ICS-CLUSTERS-31-08	TAAIKGMVSSLDPHSSYLDK	carboxyl-terminal protease [3.4.21.-]	238563023	237810682	134297049
MP-ICS-CLUSTERS-31-11A	TVELHFALGNLFRRR	tetratricopeptide repeat protein	67642911	237813292	134295082
MP-ICS-CLUSTERS-31-09	VPTLQKNAVPKLVDGA	conserved hypothetical protein	238561685	237813300	134295073
MP-ICS-CLUSTERS-31-16	ARGVRGIINFSGGLRQDLCEG	conserved hypothetical protein	67641022	237810595	134297116
MP-ICS-CLUSTERS-31-20	GETYTASVRMQLDTALMPKPF	conserved hypothetical protein	67642458	237810345	134297371
MP-ICS-CLUSTERS-31-04A	ARQFKSLEDLKGKKL	amino acid ABC transporter, periplasmic amino acid-binding protein	67640271	237812103	134295624
MP-ICS-CLUSTERS-31-13	DDGIKGMLGHFNSGTTIPASRI	putative periplasmic substrate-binding protein	67643100	237814333	134294246
MP-ICS-CLUSTERS-31-18	REKMKKLYAPFNRNHERTIYMDV	UDP-glucose 6-dehydrogenase (udg)	67642910	237813291	134295083
MP-ICS-CLUSTERS-31-15	DNNFRYSRAVLDACL	ADP-L-glycero-D-manno-heptose-6-epimerase (rfaD)	67642908	237813289	134295085
MP-ICS-CLUSTERS-31-14	VEGVRVLAGVNLPMLVRA	PTS system, mannose/fructose/sorbose (Man	67640741	237810677	134297053
MP-ICS-CLUSTERS-31-19	VPSYVYQAGLKSFDDI	ABC transporter, quaternary amine uptake transporter (QAT) family	238563533	237509058	134292111

## List of Abbreviations

BPM: Burkholderia pseudomallei
BM: Burkholderia mallei
BC: Bukholderia cepaciae
MHC: major histocompatability complex
APC: antigen presenting cell
ORF: open reading frame
HLA: human leukocyte antigen
ICS: immunogenic consensus sequence
NIAID: National Institute of Allergy and Infectious Diseases
